# Inhaled mometasone furoate for the management of refractory oral corticosteroid-dependent asthma: a case report

**DOI:** 10.4076/1757-1626-2-7770

**Published:** 2009-09-11

**Authors:** Talal M Nsouli

**Affiliations:** 1 Department of Pediatrics, Georgetown University School of Medicine, Washington DC 20007, USA

## Abstract

We report a case study of a 55-year-old white male with severe persistent refractory corticosteroid-dependent asthma receiving inhaled combination therapy with fluticasone propionate 500 μg and salmeterol 50 μg twice-daily in addition to 6-week cycles of oral corticosteroid treatment for the previous 7 months. The patient was switched to high-dose mometasone furoate delivered via a dry powder inhaler (660 μg twice-daily) for 6 weeks.

Considerable improvement from baseline in peak expiratory flow, use of rescue medication, and asthma symptoms of coughing and wheezing was observed. The patient discontinued the oral corticosteroid after 1 week of high-dose mometasone furoate treatment. Plasma cortisol value at 8 a.m. was 8.4 μg/dL (normal range, 4.3-22.6 μg/dL) at week 6.

## Introduction

Inhaled corticosteroids (ICSs) are the standard controller therapy for management of persistent asthma [1]. The majority of patients with asthma have mild-to-moderate disease, which can be controlled with maintenance therapy using ICS monotherapy. However, approximately 10% of patients are considered to have severe disease. These patients are not well controlled despite high-dose ICS and long-acting β_2_-agonist (LABA) therapy; some of these patients may even require treatment with oral corticosteroids (OCS) [2]. Occasionally, patients dependent on OCS become refractory to therapy, a condition commonly known as refractory corticosteroid-dependent asthma (RCDA). RCDA does not have a simple definition. The condition is characterized by the medication requirement for good disease control or persistent symptoms, asthma exacerbations, or airway obstruction regardless of high use of medication. Clinically, such patients may present with large variations in peak flows, rapid and progressive loss of lung function, severe but chronic airflow limitation, wide-ranging amounts of mucus production, and varying responses to corticosteroids [3]. Severe refractory asthma, including corticosteroid-dependent asthma, likely occurs in less than 5% of cases [3]. However, severe asthma accounts for a substantial proportion of total costs associated with treatment [4]. RCDA is very difficult to control; therefore, alternative approaches are necessary to improve management of patients who suffer from it. Here, a novel approach of switching a patient with RCDA on fluticasone propionate (FP) plus salmeterol (SAL) therapy to high-dose mometasone furoate delivered via a dry powder inhaler (MF-DPI) therapy was tested.

## Case presentation

A 55-year-old white male presented with RCDA. The patient had uncontrolled, severe, OCS-dependent allergic asthma and had been receiving inhaled combination therapy with FP 500 μg and SAL 50 μg twice-daily (FPS) along with OCS treatment in 6-week cycles for the previous 7 months, in addition to montelukast 10 mg daily. The FPS therapy was stopped, and the patient was switched to MF-DPI 660 μg twice-daily for 6 weeks. The patient continued OCS, montelukast, and as-needed albuterol therapy. Peak expiratory flow (PEF), coughing and wheezing, albuterol use, and OCS use were recorded at initiation (baseline) and at week 6 of MF-DPI 660 μg twice-daily treatment; plasma cortisol levels at 8 a.m. were measured at week 6.

After 6-weeks of treatment with MF-DPI 600 μg twice-daily, clinical improvement was observed when compared with baseline. PEF increased from 375 L/min to 600 L/min (Table 1). After 1 week of high-dose MF-DPI treatment, the patient discontinued use of OCS. The frequency of albuterol use decreased from 8 puffs/d to 4 puffs/d (Table 1). Cough frequency decreased, and wheezing improved from continuous to no wheezing. After 6 weeks of high-dose MF-DPI treatment, the plasma cortisol level at 8 a.m. was still within normal limits (8.4 μg/dL, normal range 4.3-22.6 μg/dL).

**Table 1:** Effects of MF-DPI on lung function, albuterol use, and cortisol levels

	Baseline (with FPS)	Week 6 (with MF-DPI 660 µg BID)
PEF, L/min	375	600
Use of albuterol, puffs/d	8	4
8 AM plasma cortisol, µg/dL^*^	ND	8.4

BID, twice-daily; FPS, fluticasone propionate and salmeterol; MF-DPI, mometasone furoate delivered via a dry powder inhaler; ND, not determined; PEF, peak expiratory flow; puffs/d, puffs per day.

*Normal range is 4.3-22.6 µg/dL.

The allergy skin prick testing (ASPT) was conducted on the volar aspect of the forearms, using disposable hypodermic needles (26 gauge) and commercially available aeroallergen solutions: pollens (trees, including elm, maple, oak, 1:20 w/v; grasses, including timothy, sweet vernal, bermuda, 10,000 BAU/mL; weeds, including ragweed, cocklebur, sheep sorrel, 1:20 w/v); pet dander (cat 10,000 BAU/mL, dog 1:10 w/v); dust mites (*Dermatophagoides pteronyssinus* and *D. farinae* 30,000 AU/mL); and molds (including *Alternaria* and *Cladosporium*, 1:10 w/v). Positive and negative control solutions were also applied (histamine phosphate 10 mg/mL and phosphate-buffered saline with 0.4% phenol, respectively). The ASPT was positive to pollen, dust mite, molds, and cat dander.

## Discussion

Other alternative therapies have also been prescribed for refractory asthma. Immunomodulating drugs such as inhibitors of tumor necrosis factor-α (etanercept) [5] and intravenous immunoglobulins [6] have been used in treating refractory asthma; however, no consistent improvement in lung function has been demonstrated. The use of other corticosteroid-sparing drugs, including oral gold and methotrexate, has also been proposed in patients who are resistant to corticosteroids, but little therapeutic effect has been demonstrated and substantial safety concerns exist with these medications [2].

In this patient with uncontrolled severe asthma, switching therapy from FPS to high-dose MF-DPI reduced symptoms, decreased use of rescue medication, and established adequate disease control allowing for discontinuation of OCS therapy. In addition, high-dose MF-DPI did not suppress the hypothalamic-pituitary-adrenal axis and had a favorable safety profile.

## Conclusion

In conclusion, high-dose MF-DPI was found to be an effective and safe treatment option for patients with RCDA who are receiving high-dose FPS and OCS therapy. The approach of switching patients with RCDA to an alternate high-dose ICS needs to be investigated in large, randomized, placebo-controlled trials.

## Abbreviations

ASPT: allergy skin prick testing; FP: fluticasone propionate; FPS: combined fluticasone propionate and salmeterol; ICS: inhaled corticosteroid; LABA: long-acting β_2_-agonist; MF-DPI: mometasone furoate delivered via a dry powder inhaler; OCS: oral corticosteroid; PEF: peak expiratory flow; RCDA: refractory corticosteroid-dependent asthma; SAL: salmeterol.

## Consent

The patient has provided consent for publication on the condition that his/her identity is protected. A signed consent form is therefore not available.

## Competing interests

Dr. Nsouli has served on speaker's bureaus for Aventis, Merck, AstraZeneca, Schering-Plough, Genentech, Teva, Sepracor, and Verus-Pharma. The author declares that he has no competing interests.
